# Lipid Nanoparticle Formulation Increases Efficiency of DNA-Vectored Vaccines/Immunoprophylaxis in Animals Including Transchromosomic Bovines

**DOI:** 10.1038/s41598-020-65059-0

**Published:** 2020-05-29

**Authors:** Eric M. Mucker, Priya P. Karmali, Jerel Vega, Steven A. Kwilas, Hua Wu, Matthew Joselyn, John Ballantyne, Darryl Sampey, Rajesh Mukthavaram, Eddie Sullivan, Pad Chivukula, Jay W. Hooper

**Affiliations:** 1 US Army Medical Research Institute for Infectious Disease, Fort Detrick, MD USA; 2 Arcturus Therapeutics, San Diego, CA USA; 3 Aldevron, Fargo, ND USA; 4 SAB Biotherapeutics, Sioux Falls, SD USA; 5 BioFactura, Frederick, MD USA

**Keywords:** Gene delivery, DNA vaccines

## Abstract

The use of nucleic acid as a drug substance for vaccines and other gene-based medicines continues to evolve. Here, we have used a technology originally developed for mRNA *in vivo* delivery to enhance the immunogenicity of DNA vaccines. We demonstrate that neutralizing antibodies produced in rabbits and nonhuman primates injected with lipid nanoparticle (LNP)-formulated Andes virus or Zika virus DNA vaccines are elevated over unformulated vaccine. Using a plasmid encoding an anti-poxvirus monoclonal antibody (as a reporter of protein expression), we showed that improved immunogenicity is likely due to increased *in vivo* DNA delivery, resulting in more target protein. Specifically, after four days, up to 30 ng/mL of functional monoclonal antibody were detected in the serum of rabbits injected with the LNP-formulated DNA. We pragmatically applied the technology to the production of human neutralizing antibodies in a transchromosomic (Tc) bovine for use as a passive immunoprophylactic. Production of neutralizing antibody was increased by >10-fold while utilizing 10 times less DNA in the Tc bovine. This work provides a proof-of-concept that LNP formulation of DNA vaccines can be used to produce more potent active vaccines, passive countermeasures (e.g., Tc bovine), and as a means to produce more potent DNA-launched immunotherapies.

## Introduction

It is now possible to rapidly determine the genomic sequence of emerging infectious disease threats, even before the microbe has been isolated. That is, nonspecific amplification and sequencing of nucleic acid can be performed when virus is difficult to propagate and/or in samples where viable virus no longer exists. This genomic information can then be used to design and synthesize candidate nucleic acid-based vaccines. The vaccines can be used as active vaccines, or as passive vaccines to produce antibody-based medical countermeasures. In order to increase the potency of plasmid DNA vaccines, we have explored the possibility of formulating the DNA using lipid nanoparticles (LNPs). The microbes targeted in this research include Andes virus (ANDV) and Zika virus (ZIKV).

ANDV is a South American, New World hantavirus and member of the *Family Hantaviridae*, *Order Bunyavirales*. The enveloped virion contains a single stranded, tripartite, negative sense RNA genome. The three segments, S, M, and L, encode the nucleocapsid protein (N), the envelope glycoproteins (G_n_/G_c_), and the RNA-dependent RNA polymerase, respectively. ANDV is rodent-borne and causes a severe disease referred to as hantavirus pulmonary syndrome (HPS) in humans. ANDV is the only hantavirus known to transmit person-to-person. The case fatality rate of this virus is >35% even in modern intensive care units. There are no vaccines or drugs approved to prevent or treat HPS^1^.

ZIKV belongs to the *Family Flaviviridae* and is mainly transmitted by *Aedes* mosquitos, but non-vector borne transmission has been reported^2^. The positive sense RNA genome of ZIKV is translated into a single polyprotein comprised of structural proteins including the precursor membrane protein (prM), envelope protein (E), capsid protein (C), and nonstructural proteins (NS1-NS5)^3^. ZIKV became a widely recognized public health threat in 2015 due to an increase in the incidence of associated complications of infection, such as fetal microcephaly if infected during pregnancy, and a trigger for Guillain-Barré syndrome^4,5^.

DNA vaccines have been constructed for both ANDV^6–8^ and ZIKV^9–12^. The ANDV DNA vaccine contains the M gene open reading frame encoding G_n_/G_c_. The ZIKV DNA vaccines contain sequences encoding the structure proteins (prME) modified with a Japanese encephalitis virus signal sequence^13^. The ANDV and ZIKV DNA vaccines have moved, or are moving, into active vaccine clinical trials^14^ and both vaccines have been utilized to develop candidate antibody-based products^15,16^. Although safe, immunogenic, and amenable to rapid transition from target sequence to prototype vaccine, the delivery of the DNA vaccine drug substance using needle and syringe is inefficient. To improve DNA vaccine immunogenicity, and to reduce vaccine dose and associated cost-of-goods, researchers have utilized methods to modify/enhance the immune response (i.e., adjuvants) or to increase delivery (e.g., electroporation, needle-free jet injection, and formulation)^17^. For example, we previously reported that disposable syringe jet injection (DSJI) could be used to increase the immunogenicity of the ANDV DNA vaccine relative to needle and syringe^6^.

Here, we demonstrate that combining DSJI with a DNA vaccine formulated with a lipid nanoparticle (LNP) carrier, which was originally developed for RNA, can further increase DNA vaccine immunogenicity in non-rodent laboratory animals including rabbits and nonhuman primates (NHPs). The LNP carrier is part of a technology known as LUNAR® (Lipid-enabled nucleic acid delivery reagent) and has been shown to be effective and safe for up to 10 mg/kg of RNA in mice^18,19^. Having found that LNP formulation of DNA could increase immunogenicity, probably by increasing DNA uptake and subsequent immunogen production, we applied the technology to a product-oriented application: improving production of fully human polyclonal antibodies in transchromosomic (Tc) bovines–animals engineered to produce human immunoglobulins (Ig).

## Results

### LNP formulation of the ANDV DNA vaccine results in a rapid, dose dependent neutralizing antibody response in vaccinated rabbits

**Figure 1:**
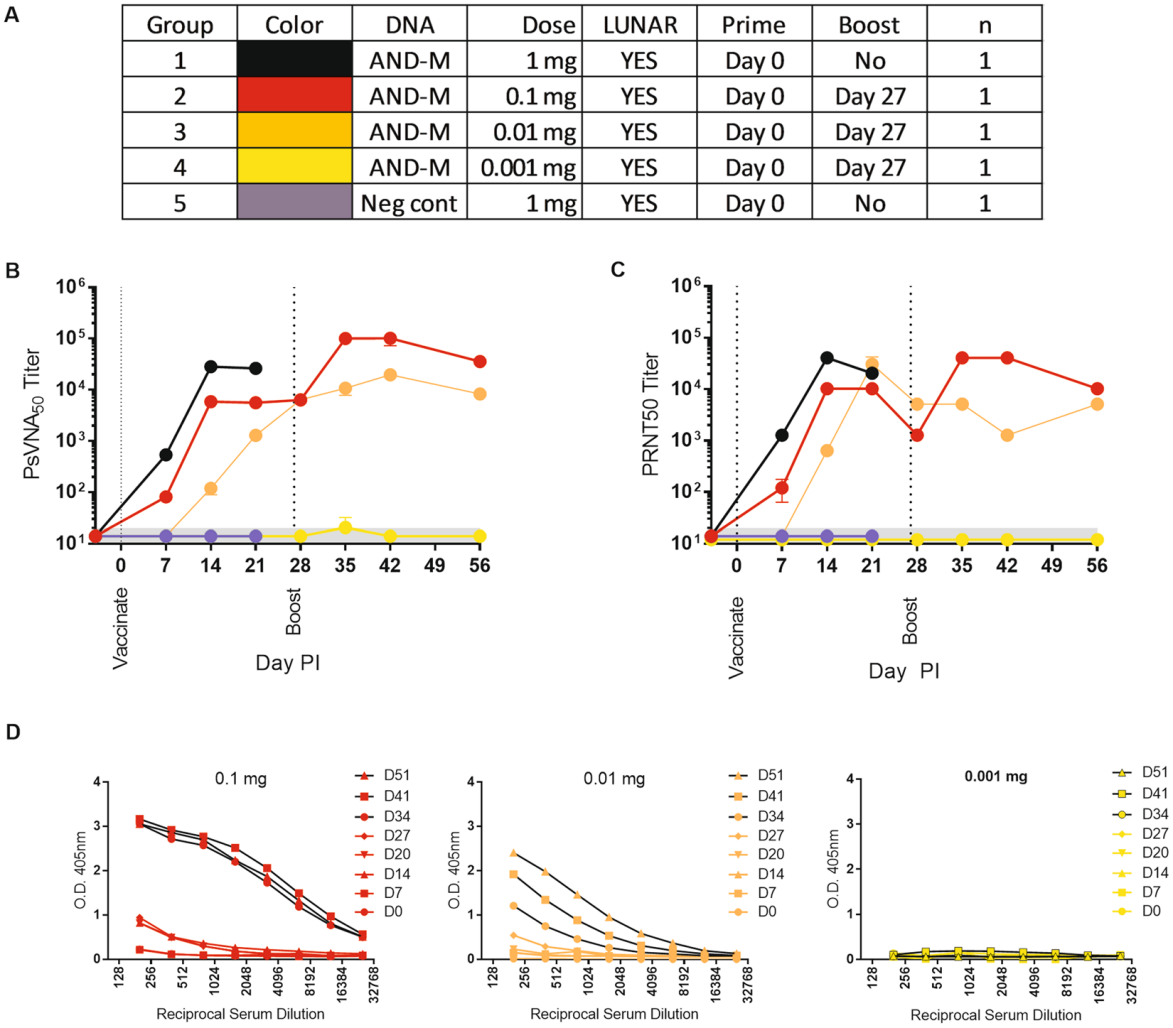
Evaluation of LNP-formulated DNA vaccine in rabbits. Rabbits were vaccinated with the ANDV DNA vaccine (AND-M) formulated with LUNAR® LNP. (**A**) Experimental design legend. **(B**) Serum samples were assayed for the presence of neutralizing antibodies by PsVNA, or **(C**) PRNT using wildtype ANDV. The limit of quantitation was a titer of 20 (grey shaded area). (**D)** ELISA were conducted to detect GnGc binding antibodies before and after the Day 27 boost.

Application of Arcturus’s LUNAR® technology results in increased delivery of mRNA into the cell and a concomitant increase in production of the encoded protein *in vitro* an *in vivo*^18,19^. We hypothesized that formulation of DNA with the same LNP would have a similar effect. To assess general tolerability and immunogenicity, we evaluated LNP-formulated and unformulated ANDV DNA vaccine in rabbits. Here we chose to do a dose-probing study; that is, a single rabbit was utilized per dose over a range of doses^20^. Decreasing concentrations of the ANDV DNA vaccine, or off-target DNA plasmid sharing the same backbone (pWRG7077), were injected intramuscularly using a DSJI (PharmaJet Stratis®) (Fig. 1A). This strategy was utilized in consultation with USAMRIID Veterinarians, Statistician, and IACUC. From the onset, it was determined that due to our lack data supporting the tolerability of our LNP formulated DNA, it was not ethically or scientifically justifiable to increase the number of animals per group. Therefore, the data generated are required to help select an acceptable (immunogenic and tolerable) dose for our next, statistically robust comparative experiment.

A neutralizing response, as determined by an ANDV pseudovirion neutralization assay (PsVNA) and a plaque reduction test (PRNT), was detected after a single injection of either 1, 0.1 or 0.01 mg of LNP-formulated DNA at our earliest evaluated time point (Day 7) (Fig. 1B,C). The magnitude of neutralization (titer) increased until Day 14, at which time the titer plateaued (PsVNA) or slightly decreased (PRNT) by Day 27. The response was dose dependent in that the highest titer after the first injection was observed in the 1 mg animal followed by the 0.1 and 0.01 mg animals, respectively, until Day 27. ELISA data demonstrated that there were detectable, albeit low, levels of binding antibodies in sera after a single injection with the 0.01 mg doses. (Fig. 1D). Animals vaccinated with a concentration of 0.001 mg of ANDV DNA vaccine or off-target DNA plasmid were negative by PsVNA, PRNT, and ELISA.

One month after the initial vaccination, animals received the same dose of vaccine as initially administered (Fig. 1A). The animal vaccinated with 1 mg died after boosting (see Discussion). An increase in titer was evident in the 0.1 mg animal using both PRNT and PsVNA assays. The 0.01 mg showed a modest increase in the PsVNA titer but no gain by PRNT (Fig. 1B,C). The ELISA data demonstrated that there was an increase in the level of binding antibodies after the 0.1 mg and 0.01 mg boosts (Fig. 1D). The animal boosted with 0.001 mg remained negative, or close to background, in all assays (Fig. 1B–D).

### Responses to LNP-formulated ANDV DNA vaccine were more rapid, of higher magnitude, and had less variability than unformulated ANDV DNA vaccine

**Figure 2:**
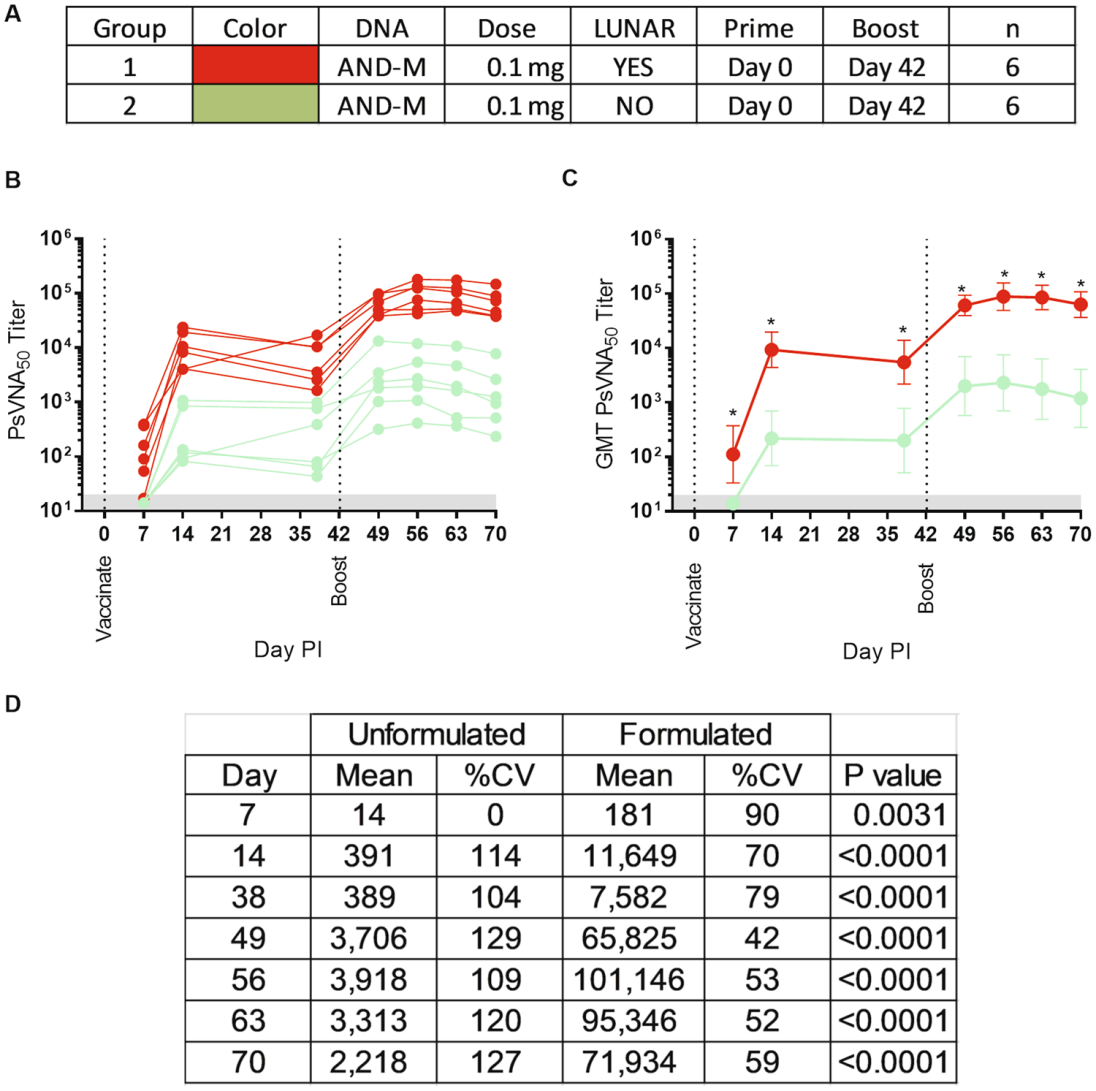
A head-to-head comparison of LNP-formulated versus unformulated ANDV DNA vaccine. Groups of six rabbits were vaccinated with the ANDV DNA vaccine (AND-M) formulated with LNP (LUNAR®), or unformulated. (**A**) Experimental design legend. **(B)** The rabbit sera from multiple time points were assayed for neutralizing activity in the ANDV PsVNA and the results graphed by individual rabbit. (**C**) Group ANDV PsVNA geometric mean titer (GMT). (**D)** Specific differences between the GMT and coefficient of variation (%CV) per sampling day are summarized. Asterisks indicate a significant difference (p-value <0.05) between means and corresponding p-values are given. Day PI = Day Post Injection.

To directly compare the effect of formulation with LNP on DNA vaccine immunogenicity, we conducted a head-to-head experiment evaluating LNP-formulated and unformulated ANDV DNA vaccine in rabbits utilizing 0.1 mg of DNA. We chose 0.1 mg based on the high immunogenicity and tolerable reactogenicity observed in the previous experiments. All rabbits received homologous boosts on Day 42 post-vaccination (Fig. 2A).

Data from PsVNA show a difference in the onset of neutralization activity (Fig. 2B,C). LNP-formulated ANDV DNA vaccine elicited neutralizing antibodies on Day 7, which was the first sample collection time point after vaccination. Unformulated ANDV DNA vaccine were all negative on Day 7 and then all positive on Day 14. Prior to boosting, the magnitude of neutralizing activity for both groups peaked on Day 14, with the mean neutralizing activity of the LNP-formulated vaccine group approximately 30 times greater than that of the unformulated vaccine group. A slight drop was noted in both groups prior to boosting. After the boost, there were increases in titers relative to the pre-boost titers. The highest titers for both groups occurred on Day 56 post vaccination and, again, the formulated DNA vaccine group had titers almost 30 times greater than the unformulated DNA vaccine. Statistically, there was a significant difference between titers at all post vaccination sampling time points (Fig. 2C,D). The LNP-formulated DNA vaccine group consistently had a lower percent coefficient of variation than the unformulated DNA vaccine group (Fig. 2D).

### LNP-formulated ANDV DNA vaccine is more immunogenic than unformulated ANDV DNA vaccine in NHPs

**Figure 3:**
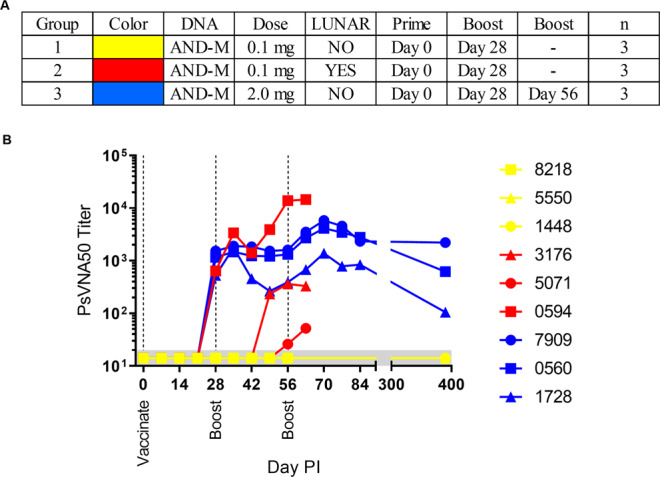
LNP-formulation of the ANDV DNA vaccine increases immunogenicity in nonhuman primates. Cynomolgus macaques were vaccinated with either 0.1 mg of LNP-formulated or unformulated ANDV DNA vaccine and boosted 28 Days later. A third group received a dose utilized in clinical studies (2.0 mg) and was boosted a second time on Day 56. (**A**) Legend. **(B**) Sera were analyzed for the presence of neutralizing antibody via a PsVNA and GMT were plotted.

To investigate whether LNP-formulation of a DNA vaccine would translate from rabbits to an animal that is more physiologically similar to humans, cynomolgus macaques were administered 0.1 mg of the ANDV DNA vaccine, either unformulated (n = 3; Group 1) or LNP-formulated (n = 3; Group 2) (Fig. 3A) via intramuscular PharmaJet injection. As a positive control, a dose and schedule identical to a published hantavirus DNA vaccine phase 1 clinical trial^21^ (2.0 mg three times at 1-month intervals) was used (n = 3; Group 3).

After a single vaccination, all animals in the 2.0 mg group were positive on Day 28 by PsVNA (Fig. 3B). Whereas only one animal of three was positive in the LNP-formulated (0.1 mg) group. All animals in the unformulated DNA (0.1 mg) group were below the limit of detection. After the first boost, there was no change in the 2.0 mg group or unformulated 0.1 mg group. Alternatively, the titer from the animal administered LNP-formulated vaccine that was positive on Day 28 increased by approximately 23-fold. This titer is approximately 5-fold higher than the highest positive control titer on Day 63 (i.e., Group 3). Furthermore, the remaining two animals within the Group 2 were positive by Day 56. For Group 1, at no time were titers above background levels.

As an aside, a second boost was administered to the 2.0 mg unformulated group only to emphasize the potential of DNA vaccines alone and to define a long duration target for our formulated test article. Unfortunately a second boost of formulated boost could be not administered due to lack of LNP formulated ANDV DNA. For the unformulated DNA vaccine, neutralizing antibody levels increased in all three animals and were still detected by both the PsVNA and PRNT at the last time point tested, approximately 1 year after the last vaccination

Together these data demonstrate that LNP-formulation of ANDV DNA can increase the neutralizing response in NHPs; however, unlike the response in rabbits, the response in macaques was highly variable.

### Increased immunogenicity of LNP formulation of DNA is not immunogen specific

To determine if the increased immunogenicity was a phenomenon of the target (ANDV M gene-encoded G_n_/G_c_ envelope glycoproteins), we tested an alternative DNA vaccine targeting ZIKV. The ZIKV DNA vaccine consists of optimized sequence encoding the ZIKV pRM/E protein fused to a JE signal sequence in the same DNA backbone (pWRG7077 plasmid) used for the ANDV DNA vaccine. This ZIKV DNA vaccine has been shown to elicit neutralizing (and protective) antibodies in rabbits and Tc bovine^15^.

**Figure 4:**
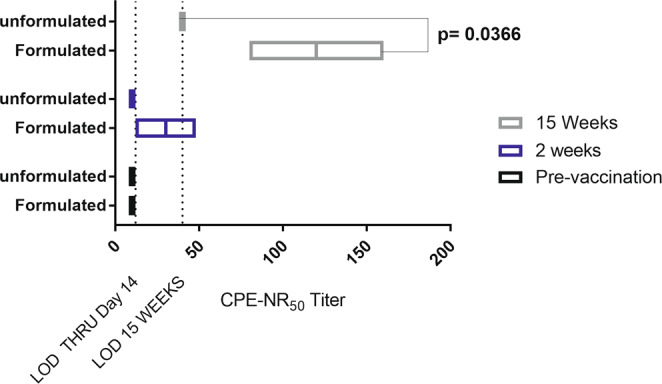
LNP-formulated ZIKV DNA vaccine is more immunogenic than unformulated vaccine at relatively low doses. Cynomolgus macaques were vaccinated with either 0.3 mg of LNP-formulated (n = 2) or unformulated (n = 3)ZIKV DNA vaccine. The dilution of sera that reduced 50% of a 100 pfu of ZIKV, determined by a reduction in cytopathic effect (CPE) as measured by neutral red uptake. LOD: limit of detection. CPE-NR50 = the reciprocal of the dilution neutralizing CPE by 50%. Significant difference (p-value <0.05) between means and corresponding p-value is given.

Cynomolgus macaques were vaccinated with either a LNP-formulated or unformulated version of the ZIKV DNA vaccine using PharmaJet Stratis®. Temporal serum samples were evaluated for neutralizing activity using a ZIKV CPE neutralization assay. A dose of 0.3 mg was used in an attempt to reduce the variability observed with the 0.1 mg dose of LNP-formulated ANDV DNA vaccine in NHPs. After two weeks, neutralizing activity to ZIKV was detected in the two NHPs receiving LNP-formulated vaccine. A third NHP that received the LNP-formulated vaccine unexpectedly became ill and was euthanized a few days after vaccination. Pathology suggested a pre-existing condition, indicating kidney damage and immunosuppression. Therefore, the data from this animal was not included and all statistics were performed in the absence of that data (Fig. 4). The two animals vaccinated with the LNP-formulated vaccine had titers of 80 and 160 at 15 weeks, whereas the three animals receiving unformulated vaccine were below detection at all time points (Fig. 4). There was a statistical difference between LNP-formulated (n = 2) and unformulated (n = 3) groups at week 15 (p = 0.0366), but not at week 2 (p = 0.0805) or pre-vaccination (p = >0.9999) (Fig. 4). These data demonstrate that the increased immunogenicity associated with the LNP-formulated DNA vaccines is not vaccine target specific.

### *LNP-formulated DNA results in increased production of encoded gene products*

**Figure 5:**
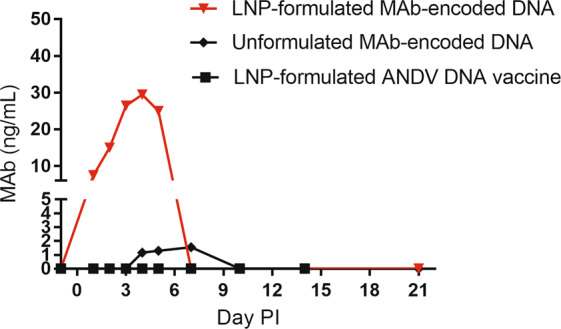
LNP-formulation of DNA plasmid results in an increase in the amount of protein produced after intramuscular injection. Rabbit (n = 1 per group) were injected with 1.0 mg of two different DNA plasmids sharing a common backbone (pWRG7077), encoding either an anti-poxvirus human chimeric monoclonal antibody (MAb-encoded DNA) or the ANDV DNA vaccine. MAb-DNA was either formulated with LUNAR® LNP or diluted in PBS prior to injection. The ANDV DNA vaccine was LUNAR® LNP-formulated. An immunogen specific ELISA was utilized to quantitate temporal concentrations of human chimeric monoclonal antibody (c7D11) in the serum.

It is possible that the increased immunogenicity observed with the LNP-formulated DNA vaccines was a consequence of increased efficiency of DNA delivery to host cells and a resultant increase in production of target antigen. To test the possibility that LNP-formulated DNA could enter cells more efficiently than unformulated DNA, we conducted an experiment in cell culture. Individual lots of LNP-formulated DNA were added to 293 T cells *in vitro* and compared with approximately the same concentration of unformulated plasmid DNA. As a control, the plasmid DNA was also transfected into cells using the commercial transfection reagent FuGENE 6. *In vitro*, uptake of the LNP-formulated DNA as measured by expression of target proteins in cells was much more efficient than DNA alone, and approached the efficiency of FuGENE 6 (Table S1). To confirm if increased production of DNA-encoded protein does occur, we used a plasmid encoding the heavy and light chain monoclonal antibody (MAb) sequences of the mouse-human chimeric, poxvirus specific antibody, c7D11^22^. Because it is secreted and has a specific ligand, we could directly assess the amount of protein being produced (*in vitro* or *in vivo*) using a quantitative ELISA^22^. After testing the functionality of the DNA construct *in vitro* by transfecting cells with a commercial reagent (Fig. S1A), we confirmed that LNP-formulated DNA was also capable of transfecting at least two different cell lines, by measuring MAbs secreted into the supernatant (Fig. S1B). There was no detectable binding activity in the supernatants of cells transfected using DNA alone (e.g., no commercial transfection reagent, or LNP-carrier). Next, we tested whether or not we could increase protein production *in vivo*. To do this, rabbits were injected with 1 mg of LNP-formulated MAb-encoding DNA, 4 mg of MAb-encoding DNA in PBS, or 1 mg of LNP-formulated negative control (ANDV DNA vaccine). Sera were collected and immunogen specific ELISAs were performed to detect the level of secreted c7D11 protein (Fig. 5). Only a single rabbit was injected with LNP formulated vaccine due to ethical/animal welfare concerns (see section titled “*LNP formulation of the ANDV DNA vaccine results in a rapid, dose dependent neutralizing antibody response in vaccinated rabbits”* and related topic in the Discussion). The rabbit receiving LNP-formulated MAb-encoding DNA had detectable levels of antibody approximately one day after injection and peaked at almost 30 ng/mL on Day 4. Values were below the limit of quantitation from the next sampling day (Day 7) and every sampling day thereafter. Antibody from the injection of unformulated MAb-encoding DNA was not detected until Day 4 and peaked on Day 7 in all 4 rabbits (mean of 1.5 ng/mL). On day 10 and subsequent sampling times, MAb levels were below the level of quantitation in sera from rabbits injected with MAb-encoding DNA in PBS.

Although the circumstances dictated that statistically significant numbers could not utilized, these data, along with *in vitro* data, do support the possibility that LNP-formulation of DNA results in increased production of target antigen and resultant increased immunogenicity. Furthermore, these data also show the potential of LNP-formulation for DNA-vectored active immunoprophylactic applications.

### Reactogenicity of LNP-formulated DNA vaccines in rabbits and NHPs

**Figure 6:**
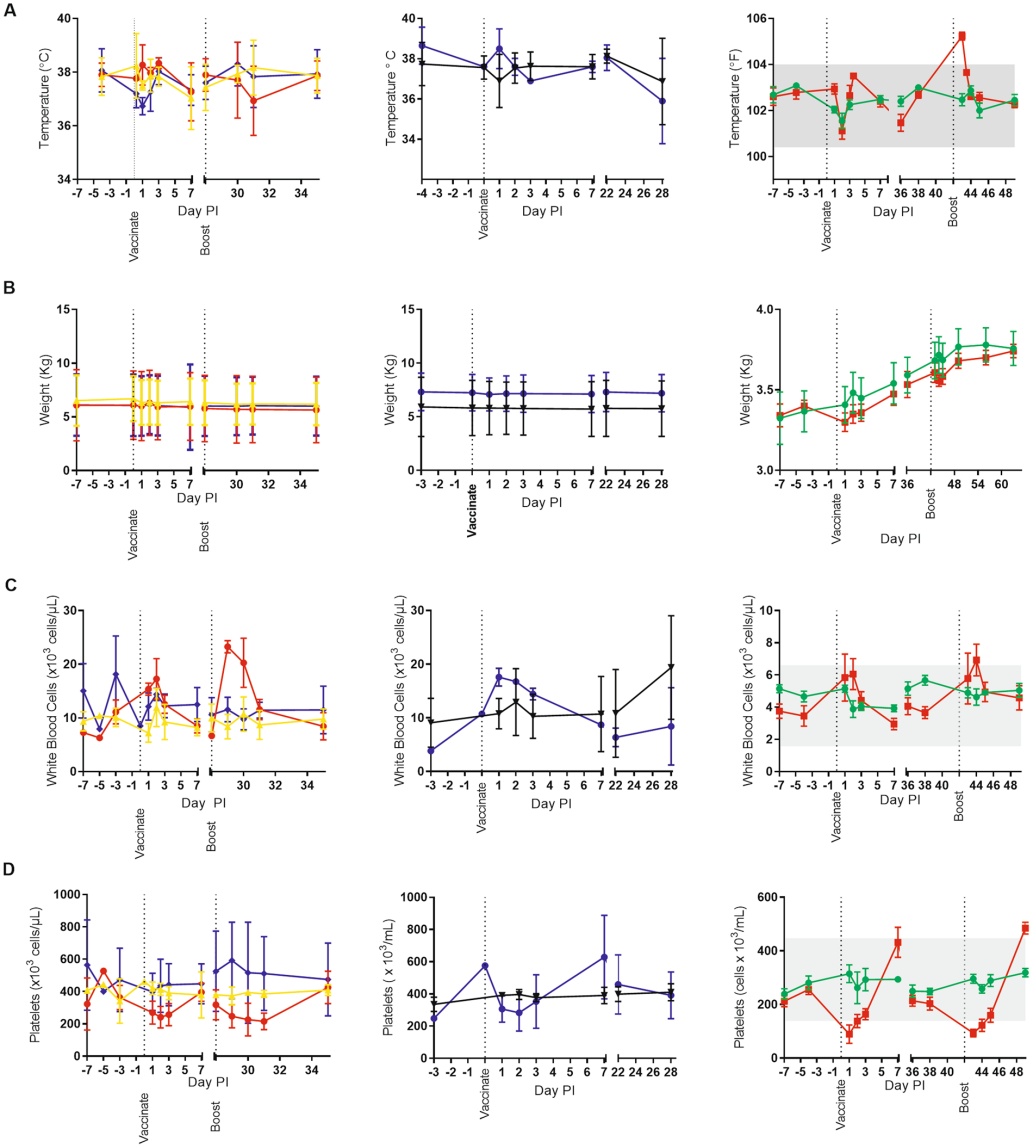
Reactogenicity of LNP-formulated DNA vaccines in rabbits and nonhuman primates. Pre- and post-vaccinations, body temperatures (**A**), body weight (**B**) and blood hematology (**C,D**) data were collected. The columns are as follows (from left to right) ANDV DNA vaccine in nonhuman primates, ZIKV DNA vaccine in nonhuman primates, and ANDV DNA vaccine in rabbits. Group identifications are as followed for the first column: red circles = 0.1 mg formulated (n = 3), yellow triangles = 0.1 mg unformulated (n = 3), and purple diamonds = 2.0 mg formulated (n = 3); for the second column: purple circles = 0.3 mg formulated (n = 2), and black triangles = 0.3 mg unformulated (n = 3); for the last column: red squares = 0.1 mg formulated (n = 6), and green circles = 0.1 mg unformulated (n = 6). Mean and standard deviation are provided for nonhuman primate data. Mean and standard error of the mean are provided for rabbit data.

**Figure 7:**
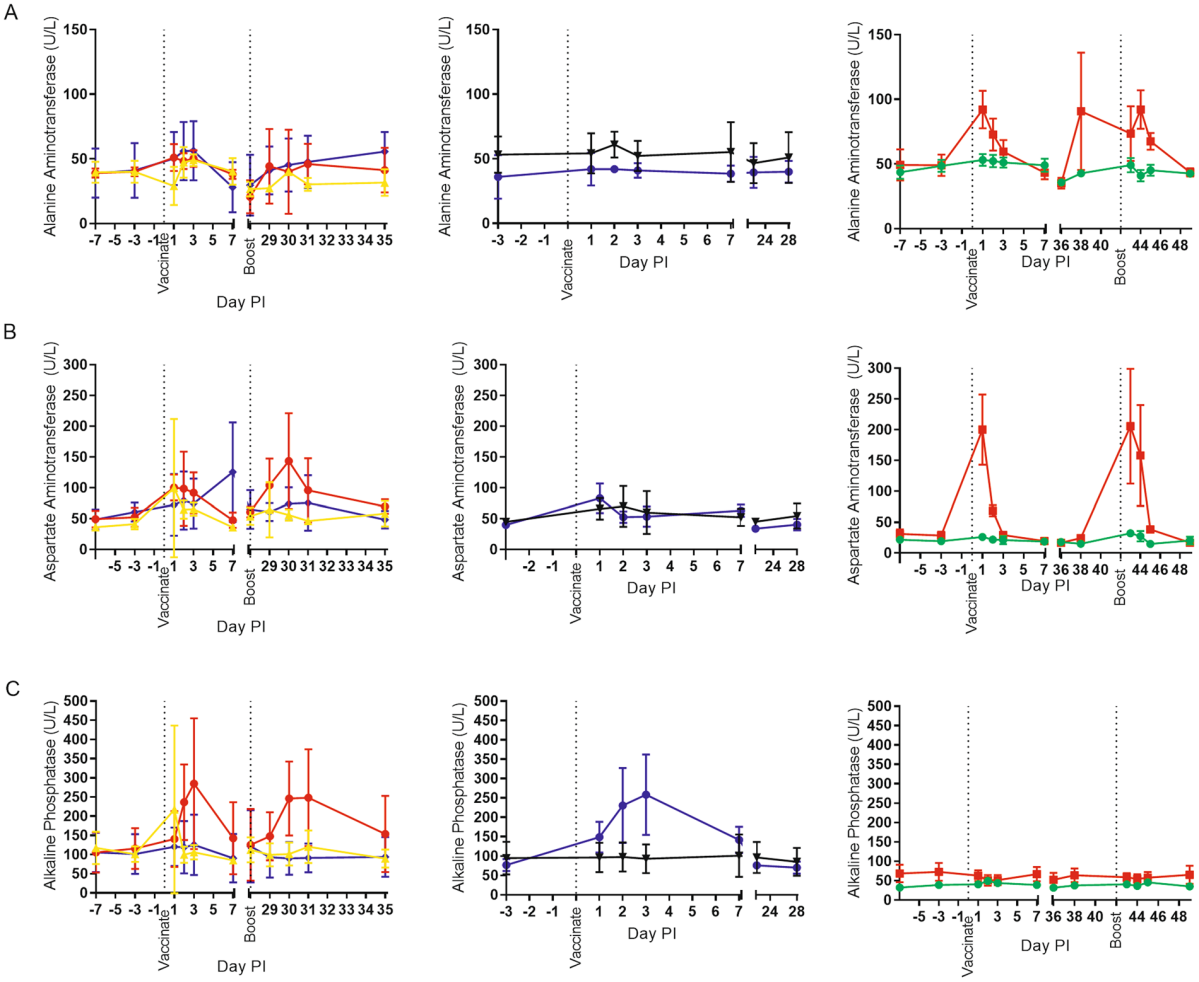
Serum Chemistry of LNP-formulated DNA vaccines in rabbits and nonhuman primates. Pre- and post-vaccination sera were collected. Chemistries were performed. Key analytes are shown including: (**A**) alanine aminotransferase, (**B**) aspartate aminotransferase, and (**C**) alkaline phosphatase. The columns are as follows (from left to right) ANDV DNA vaccine in nonhuman primates, Zika virus DNA vaccine in nonhuman primates, and ANDV DNA vaccine in rabbits. Group identifications are as followed for the first column: red circles = 0.1 mg formulated (n = 3), yellow triangles = 0.1 mg unformulated (n = 3), and purple diamonds = 2.0 mg formulated (n = 3); for the second column: purple circles = 0.3 mg formulated (n = 2), and black triangles = 0.3 mg unformulated (n = 3); for the last column: red squares = 0.1 mg formulated (n = 6), and green circles = 0.1 mg unformulated (n = 6). Mean and standard deviation are provided for nonhuman primate data. Mean and standard error of the mean are provided for rabbit data.

Although formulated DNA was generally well tolerated in our studies, rabbits did exhibit either physical signs (1.0 mg dose) and/or chemical and hematological changes. Because of this, we designed our experiments to clinically monitor both rabbits and NHPs. More specifically, we collected body weights and temperatures, and clinical chemistry and hematology data previous to, and post-vaccinations (Figs. 6 and 7). In an extreme case, a rabbit administered 1.0 mg of LNP-formulated ANDV DNA vaccine died a few days after boosting (Fig. 1).

In terms of body temperature, NHPs vaccinated with LNP-formulated DNA vaccine had no apparent fever or temperature changes, similar to those animals receiving unformulated DNA (Fig. 6A). Body temperatures of rabbits receiving LNP-formulated vaccine were consistent with the unformulated group after the primary vaccination. A brief increase in temperature was noted 24 hours after the boost vaccination, but normalized approximately 4 hours later (Fig. 6A). There were no differences in body weight between respective groups of NHPs or rabbits (Fig. 6B).

In terms of basic hematology, there were changes in white blood cells and platelets associated with LNP-formulated vaccine (Fig. 6C,D). Slight to moderate increases in white blood cells tended to occur within 24 hours of LNP-formulated vaccine administration and normalize by 7 days after the injection. This was consistent between animal species and vaccination status (i.e., boosts). A breakdown of the white blood cell shows a restructuring of WBC population as the overall increase in WBC is mainly due to neutrophilia (and to a lesser extent, an increase in monocytes), but there is also a slight decrease in lymphocytes (Fig. S2). A decrease in platelets after LNP-formulated DNA vaccinations in both rabbits and NHPs occurred, although it is more apparent in the rabbit data (Fig. 6D). Platelet values tended to normalize and/or exceed baseline levels by approximately 7 days after vaccination and/or boosting. Unformulated DNA vaccine had little/no effect on platelet counts (Fig. 6D).

For serum chemistries, NHPs and rabbits had some aberrations after injection of formulated DNA. Rabbits had a consistent increase in alanine aminotransferase (ALT) and aspartate aminotransferase (AST) within 24 hours of injection that returned to basal levels 78 hours post injection. In NHPs, the effect of formulation was a little less clear given the variability of the analytes between animals (Fig. 7A,B). More definitive changes were noted for alkaline phosphatase (Alk Phos) in NHPs (Fig. 7C). Again, the magnitude of the change was specific to individual NHPs and not dose dependent. For the NHPs that were boosted (LNP-formulated ANDV DNA vaccine), the magnitude of the change in Alk Phos was consistent with the initial vaccination. In contrast, rabbit Alk Phos levels tended to remain relatively unchanged (Fig. 7C).

### Translational application of LNP-formulated DNA to induce antigen specific human antibodies in Tc bovine

**Figure 8:**
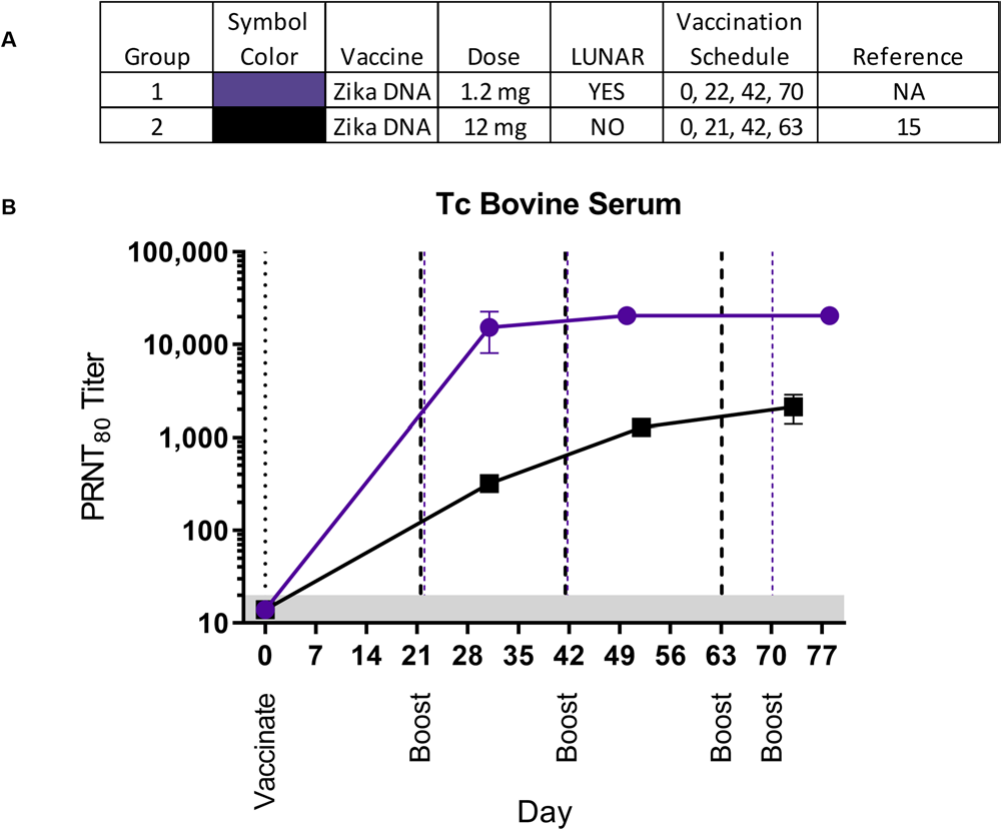
A single animal was vaccinated with 1.2 mg of LNP-formulated Zika virus DNA vaccine. Sera were collected and evaluated in a Zika virus PRNT. (**A**) Legend. (**B**) Mean PRNT80 titers were plotted with standard deviations. For comparison, published work showing the PRNT80 titers from a Tc bovine vaccinated with the same DNA vaccine unformulated and at a dose of 12 mg per vaccination is shown^15^. PRNT80 = reciprocal of the highest dilution resulting in an 80% reduction in plaques relative to no antibody control. The limit of quantitation in this assay was 40 (grey shaded area). Blue and black dashed lines indicate day of boosts for LUNAR® formulated DNA and unformulated DNA, respectively.

Having found that LNP-formulation could increase DNA vaccine immunogenicity, we applied this technology to the production of polyclonal human IgG in Tc bovine for use as a passive immunotherapy. We had previously shown that unformulated ZIKV virus DNA could be used to produce high-titer anti-ZIKV neutralizing antibodies that were protective in rodent models of ZIKV disease^15^. Here, we used the same ZIKV DNA vaccine but, because we did not know the tolerability of the LNP-formulated vaccine in TC bovine, the dose was approximately 10-fold less DNA than the unformulated DNA previously used. A single animal was utilized given the nature and rarity of TC bovine. After two vaccinations, the PRNT80 titers were>10,000 (Fig. 8). At every time point sampled, titers were more than 10 times greater than the titers previously obtained from the administration of 10 mg (per vaccination) of unformulated vaccine.

## Discussion

The research reported herein revitalizes the pragmatism and potential utility of DNA vaccine technology. Using two different DNA vaccines in three different animal species we found that it was possible to decrease the dose of DNA by an order of magnitude and still increase the levels of neutralizing antibodies by formulating the DNA.

Historically, the typical dosing schedule for hantavirus DNA vaccines has involved delivering milligrams of DNA three times at 1-month intervals. For example, in humans doses of 2.0 mg delivered by intramuscular electroporation three times at 1-month intervals were shown to elicit anti-Hantaan virus and anti-Puumala virus neutralizing antibodies in a Phase I clinical trial^21^. Similarly, rabbits dosed with 2.0 mg/vaccination of an ANDV DNA vaccine delivered by PharmaJet Stratis® three times at 1-month intervals resulted in neutralizing antibody responses, PsVNA80 GMT of 871^6^. Here, utilizing 20-fold less DNA, LNP-formulated ANDV DNA vaccine delivered by PharmaJet Stratis® as a single vaccination induced neutralizing levels that were almost 2-fold higher (PsVNA80 GMT 1548) and requiring only 14 days to do so (Fig. 2C). More impressively, the final titers in this experiment were 25–40 times higher than the titers reported in the literature despite the fact that the amount of LNP-formulated DNA was only 0.1 mg/vaccination and only two vaccinations were administered. To more directly demonstrate the benefit of LNP-formulation of DNA, we directly compared LNP-formulated and unformulated ANDV DNA vaccine in rabbits at the 0.1 mg/vaccination dose and found a significant increase in neutralizing antibodies at every time point with an average increase of 23-fold. The %CV was significantly lower at every time point indicating that the LNP-formulated vaccines reduce the variability of the immune response.

Two LNP-formulated DNA vaccines were tested in NHPs. We observed increased immunogenicity when LNP-formulated DNA was used for both ANDV and ZIKV DNA vaccines; however the absolute differences were difficult to assess because the non-LNP-formulated vaccines were nonimmunogenic when administered at an equivalent dose of DNA. For the ANDV DNA vaccine, the dose used was 0.1 mg/vaccination and for the ZIKV DNA vaccine the dose was 0.3 mg/vaccination. All of the NHPs vaccinated with the LNP-formulated DNA vaccines developed neutralizing antibodies, but unlike the rabbits, the responses in macaques were highly variable, especially animals receiving the lower dose of ANDV DNA vaccine. It is possible the variability is due to inconsistent delivery to the muscle using PharmaJet Stratis®; however, this was not the case for the rabbits where the same device was used and the muscle size was similar. Another possibility is that the 0.1 mg dose of LNP-formulated DNA used is at, or around, the minimum effective dose for NHPs. Further dose-ranging studies are required to determine if the variability in immunogenicity can be reduced by adjusting vaccine dose and/or schedule. It is likely the dose responses will be species-specific and more individualistic in primates and humans given their heterogenic backgrounds.

Others have used rhesus macaques vaccinated with 1–5 mg of different ZIKV DNA vaccine constructs (using different plasmid backbones and sequences from different strains of ZIKV), administered by needle/syringe or PharmaJet Stratis®, and measured neutralization using a slightly different assay^10,12^. Because of these differences, it is difficult to make a direct comparison of our data. With that in mind, we detected neutralizing antibody after a single injection (Day 14) and neutralizing levels persisted for at least 15 weeks, whereas a boost was required to detect neutralizing antibody when a nonformulated vaccine was used (e.g., requiring a total of 10 mg of unformulated DNA)^10^. Dowd, after a single injection of 1 mg to 4 mg of ZIKV DNA delivered via PharmaJet Stratis®, reported similar seroconversion results (100%) to those we report here^12^.

One LNP-formulated DNA vaccine was tested in a Tc bovine. The same ZIKV DNA vaccine and DSJI delivery device (PharmaJet Stratis®) previously used to vaccinate a Tc bovine was used. We found that administering 10x less LNP-formulated DNA produced approximately 20-fold higher neutralizing antibody against ZIKV virus in the Tc bovine. Again, two vaccinations (a total of 2.4 mg) of LNP-formulated DNA was more potent than 4 vaccinations (40 mg) of unformulated ZIKV DNA vaccine. An obvious limitation of the Tc bovine data is the small sample size. It is important to note that several studies using Tc bovine have demonstrated that the responses to vaccination are very consistent animal-to-animal and it is highly unlikely that the 20-fold increase in neutralizing antibody levels despite using 10-times less DNA is unrelated to the LNP-formulation.

A potential limitation of our studies is that we did not evaluate the protective efficacy of the ANDV or ZIKV DNA vaccines in animal models. In previous studies we have demonstrated that passive transfer of anti-ANDV or anti-ZIKV neutralizing antibodies produced in rabbits, NHPs, and Tc bovine vaccinated with unformulated DNA vaccines was sufficient to protect in animals models of HPS and ZIKV disease^15,16^. The studies described herein show the positive impact that LNP formulation of DNA has on a specific correlate of disease protection, i.e., neutralizing antibody for ANDV and ZIKV disease.

Since LUNAR® LNP was originally developed for RNA moieties and not DNA constructs, it was incumbent that we postulate a general mechanism underlying the observed increase in DNA vaccine potency. We have shown that the LUNAR® LNP formulation increases the effective uptake of DNA into cells *in vitro* (Table S1 and Fig. S1). Unformulated DNA vaccine produces no signal when used to “transfect” cell monolayers whereas LNP-formulated DNA vaccine transfects cells with a similar efficiency as commercial transfection reagent. This carried forward *in vivo*. By injecting a plasmid encoding a monoclonal antibody targeting orthopoxviruses, we were able to confirm more effective uptake of DNA based on the increased levels of monoclonal antibody produced and detected in the serum, with the caveat that the data was obtained in a single animal. Given the magnitude of difference between the animal receiving formulated material and the four rabbits that were administered unformulated, at the same time balancing ethical considerations, it was not scientifically justifiable to repeat the experiment at this dose. Therefore, we only address the qualitative nature of the data relative to the *in vitro* work experiments. Although, future experiments should look at decreases in the dose to further evaluate the impact of LNP formulation pertaining to vectored immunoprophylaxis (i.e., *in vivo* MAb gene transfer). As an aside, it is worth noting the rapid generation (and relative high early concentration) of plasmid-derived MAb being produced in a non-rodent (i.e. rabbits) using a FDA-510k cleared device (PharmaJet Stratis®) has never been reported, although others have used electroporation to deliver plasmid-launched MAbs in mainly mice^23–26^, but also in sheep^26^. Since we utilized a non-homologous human antibody, it is likely the dramatic decrease in circulating MAb level after 5 days is due to clearance of the foreign protein by the immune system of the rabbit. We predict levels of MAb in the serum would continue to rise if the MAb constant regions were homologous to the host animal. Future studies should further explore LNP formulation of DNA to enhance vector mediated immunoprophylaxis.

It is difficult to affirmatively state that the enhanced immunogenicity is exclusively associated with uptake of LNP-formulated DNA and increased levels of target protein expression. We know that nucleic acids are inherently immunostimulatory within cells, and, in the absence of formulation, DNA can still be immunogenic. Increased uptake of DNA could result in a globally stronger immunostimulatory response. This response, although not directly related to the DNA encoded target, could amplify the immunogenicity. For most gene therapies, off-target immunostimulatory effects could be considered detrimental to desired effect of the treatment. On the other hand, for vaccines, it may be welcomed assuming stimulatory effects are tolerable to the host. It is also possible that the combination of DNA and the LNP triggers an “off-target” response similar to cationic lipoplex^27–29^. Alternatively, contaminants from DNA production (bacterial derived) may also stimulate cellular responses^30,31^.

We saw brief, transient changes in a small number of chemistry and hematology analytes when LNP-formulated DNA, but not unformulated DNA, was injected. These effects varied slightly between species. Specifically, ALK Phos abnormalities were more obvious in NHPs whereas ALT and AST changes were more prominent in rabbits. LNP-formulated RNA has not been reported to significantly alter ALT or AST suggesting that the DNA, or contaminant in the DNA preparation, in combination with LNP carrier was responsible for the aberrations. Rabbits had increased rectal temperatures 24 hours after the boost, but they quickly subsided when measured less than 4 hours later (Fig. 6). Unlike rabbits, we did not observe any increase in temperature in the NHPs. Although we did not directly measure, it is likely that the immunostimulatory effect of the LNP-formulated plasmid DNA, or contaminant, is responsible for the aberrant chemistry and hematology^28,29^. A proinflammatory cytokine induction after vaccination may also explain the increase in neutrophils and monocytes. This phenomena was common to both NHPs and rabbits in our experiments. The potential mechanism(s) for immunostimulation and reactogenicity are hypothetical and require empirical testing. Defining the mechanism of immunostimulation/toxicity will facilitate the refinement of this technology.

Because of the acceptable tolerability, we were able to immediately use LNP-formulated DNA for a real-world application involving the production of human IgG in Tc bovine. Tc bovine vaccinated with LNP-formulated DNA did not develop any adverse events, and the level of the neutralizing antibody response is several-fold higher than achieved previously. This increased level of neutralizing antibodies in the plasma should translate into more potent candidate Tc bovine-derived IgG products in the future.

Additional research is needed to investigate the feasibility of using LNP-formulated DNA for active vaccines in humans. Potential safety issues related to LNP-formulated plasmid DNA need to be addressed and, if necessary, overcome. Other issues related to vaccine development such as stability of LNP-DNA at different storage conditions, dosing, schedule, characterization of the immune responses, duration of immunity, and finally, efficacy must also be assessed for this novel vaccine approach.

## Materials and Methods

### DNA plasmids

ANDV DNA vaccine and ZIKV DNA vaccine have been described elsewhere^6^ and^15^, respectively and were produced at Aldevron. Similar to the ANDV and ZIKV DNA vaccines, pWRG7077 plasmid was utilized as the backbone for the construction of human monoclonal antibody vectors (pWRG/c7d11 (H + L)). The heavy chain and light chain sequence (including the signal sequence) were cloned into a unique NotI and BglII site of separate plasmids. The plasmid containing the light chain was then PCR amplified with forward primer GCA GGT TCT AGA CGA CAA TAT TGG CTA TT and reverse primer AAG CAA TAC ATG TGT CGA GCT GT so as to introduce XbaI and PciI restriction sites and amplify the CMV promotor, light chain and through the Xba1 restriction site present on the plasmid. The fragment was electrophoresed, band excised and digested with PciI and XbaI. The plasmid containing the heavy chain insert was digested with Xba1 and PciI, gel purified, dephosphorylated, and ligated with the PCR fragment using common techniques. Sequences for light and heavy chain antibodies are available in supplement (Fig. S3). For c7d11 (heavy and light chain) human trypsinogen-2 signal peptide, was utilized. Target sequences were optimized (*Homo sapien*) and synthesized by Genewiz, LLC.

### Plasmid LUNAR® formulation

Using a proprietary lipid delivery technology platform, LUNAR® technology, Arcturus Therapeutics manufactured LUNAR® containing plasmid DNA. The formulations were prepared by mixing of lipids in ethanol with an aqueous phase containing plasmid DNA, using a Nanoassemblr microfluidic device, DNA was dissolved in citrate buffer (pH 3.5). Lipids were dissolved in ethanol. The molar percentage ratio for the constituent lipids is 58% ATX (proprietary ionizable amino lipids), 7% DSPC (1,2-distearoyl-sn-glycero-3-phosphocholine) (Avanti Polar Lipids), 33.5% cholesterol (Avanti Polar Lipids), and 1.5% DMG-PEG (1,2-Dimyristoylsn-glycerol, methoxypolyethylene glycol, PEG chain molecular weight: 2000) (NOF America Corporation). At a flow ratio of 1:3 ethanol:aqueous phases, the solutions were combined in the microfluidic device (Precision NanoSystems). The total combined flow rate was 12 mL/min, per microfluidics chip. The mixed material was then diluted with phosphate buffer after leaving the micromixer outlet. The diluted particles were purified by dialysis in Hepes buffer (pH 7.4) containing sucrose using regenerated cellulose membranes (SpectraPor Tube-A-Lyzers, Spectrum Laboratories). A total of 200 diavolumes were exchanged, effectively removing ethanol and ensuring complete buffer exchange. The LNPs were then concentrated using ultra-spin centrifugal filter units (Amicon Ultra Centrifugal Filter Units, EMD Millipore). Particle size was determined by dynamic light scattering (ZEN3600, Malvern Instruments). DNA content and encapsulation efficiency was determined using ribogreen assay. Encapsulation efficiency was calculated by determining unencapsulated plasmid DNA content by measuring the fluorescence upon the addition of ribogreen (Molecular Probes) to the particles (Fi) and comparing this value to the total plasmid DNA content that is obtained upon lysis of the particles by 1% Triton X-100 (Ft), where % encapsulation = (Ft − Fi)/Ft × 100.

### Viruses and cells

ANDV strain Chile-9717869 and ZIKV strain ArD 41525 propagation and characterization have been previously reported^6^ Vero E6 cells (ATCC CRL-1586 were maintained in Eagle’s minimal essential medium with Earle’s salts (EMEM) containing 10% fetal bovine serum (FBS), 10 mM HEPES pH 7.4, and antibiotics (penicillin [100 U/mL], streptomycin [100 µg/mL]) (cEMEM) at 37 °C in a 5% CO_2_ incubator. For ZIKV neutralization assays, Vero 76 cells (ATCC CRL-1587) were maintained in minimal essential media (MEM) containing 10% FBS at 37 °C in a 5% CO_2_ incubator.

### ANDV pseudovirion neutralization assay

Andes virus pseudovirions (ANDV PsV) were produced, titrated, and assays conducted as described^6^. Briefly, PsV were incubated with heat inactivated serum in the presence of 10% guinea pig complement overnight at 4 °C. The mixture was then added to Vero 76 cells and incubated for 18–24 hours. The media was then removed, cells lysed and bioluminescence captured using a Tecan microplate reader. Data was analyzed using Graphpad Prism where IC50s were interpolated and reported as PsVNA50 titers.

### ANDV plaque reduction neutralization test

Vero E6 were used to perform plaque-reduction neutralization tests (PRNT) as previously described^32^. The PRNT_50_ is reported as the reciprocal of the highest serum dilution reducing the number of plaques by 50% relative to control wells that were untreated. The limit of detection in the PRNT is a titer of 20.

### ANDV PsV-based ELISA

PsV-based ELISAs were done similarly to what as previously described^33^ Briefly, PsV were coated onto a 96-well high-binding microtiter plates (Costar) at 10,000 flourescent focus units/well at 4 C in sodium carbonate buffer pH 9.6. The plates blocked before adding rabbit sera samples that were initially diluted 1:200 and were then serially diluted 2 fold. After thorough washing the plates were incubated with a 1:2000 dilution of a HRP goat anti-rabbit secondary (KPL). The plates were developed with TMB (KPL). The endpoint titer was determined as the highest dilution that had an optical density greater than the mean optical density for serum samples from negative-control wells plus 3 standard deviations.

### L1-specific ELISA

Quantitative ELISA assays were performed to determine the level of human antibody specific to poxvirus antigen, L1. These assays were performed as described^22^.

### ZIKV neutralization assay

Using 96 well plates, serum was heat inactivated (56 °C for 30 minutes) and diluted in MEM containing 5% heat inactivated fetal bovine serum (FBS). ZIKV was added to each well to a final concentration of 100 PFU/well. The samples were then incubated for 1 hour at 37 °C. Once the samples were removed, a final concentration of 1 × 10^4^ Vero cells were added to each well and returned back to the incubator. After 4 days, a 1:50 dilution (in MEM with 5% FBS) of neutral red was added to each well and placed back into the incubator for at 2 hours. The plates were decanted, and washed/decanted twice with PBS (pH 7.4). An 1:1 mix of 2% glacial acetic acid and 96% ethanol was made, of which 150uL was added per well. The plates were shaken for 10–20 minutes at 250 rpm before absorbance data (540 nm) were obtained using a Tecan reader. Graphpad Prism was used to normalize the data based on positive and negative control wells. The reported titer was based off the last dilution to yield ≤ 50% values.

### Animals

#### Rabbits

Adult female New Zealand White rabbits (*Orycytolagus cuniculus*) were purchased from Charles River Laboratories. Weights ranged from approximately 3 to 4 Kg at the time of injections.

#### NHPs

Adult male and female cynomolgus macaques (*Macaca fascicularis*) from USAMRIID’s colony (Origin: China or Cambodia) were utilized on this study. Weights of NHPs ranged from 3 to 10 Kg.

#### Tc bovines

All experimental protocols related to production of Tc bovines, immunization and sample collections described in this study were reviewed and approved by the Institutional Animal Care and Use Committee (IACUC) at SAB Biotherapeutics Inc. All experiments were performed in accordance with the approved guidelines for animal care and management of research projects.

Tc bovines were produced as previously described^16,34–36^. The Tc bovines used in this study are homozygous for triple knock-outs in the endogenous bovine immunoglobulin genes (*IGHM* − / − *IGHML1* − / − *IGL* − / − ) and carry a human artificial chromosome (HAC) vector labeled as isKcHACD. This HAC vector consists of human chromosome 14 fragment, which contains the entire human immunoglobulin heavy chain locus except that the IGHM constant region remains bovine and the key regulatory sequences were bovinized; and human chromosome 2 fragment, which contains the entire human immunoglobulin k light chain locus^16,34–36^. Tc bovines were produced by using genetically engineered and cryobanked fibroblast cells as chromatin donors via a proprietary chromatin transfer (CT) procedure.

Tc bovine hyperimmunization and sample collection. One Tc bovine (#2227) was immunized with unformulated ZIKV virus DNA vaccine at 12 mg per animal per vaccination by using the PharmaJet Stratis® IM injection device as previously described (14). Another Tc bovine (#2339) was immunized with LNP formulated ZIKV virus DNA vaccine at 1.2 mg per vaccination by using the PharmaJet Stratis® IM injection device. #2339 was vaccinated 4 times at 3 to 4-week intervals.

Prior to the first vaccination (V1), a volume of pre-vaccination plasma was collected from each study Tc bovine to be used as negative control. Up to 2.1% of body weight of hyperimmune plasma per animal per time point was collected from immunized Tc bovines for anti- ZIKV fully human polyclonal antibody production on days 8, 11 and 14 post each vaccination starting from the third vaccination (V3) to the fourth vaccination (V4). Plasma was collected using an automated plasmapheresis system (Baxter Healthcare, Autopheresis C Model 200). Plasma were stored frozen at −20 °C for future use.

### DNA administration

All intramuscular injection(s) (IM) were performed utilizing a PharmaJet Stratis® device in a volume of 0.5 mL. NHPs and rabbits were anesthetized before the tricep or lateral thigh, respectively, were clipped and subsequently injected (IM) with the test material. For Tc bovine, each vaccine was administered with one injection of 0.3 mg behind each ear and inner thigh (for 0.3 mg per site; total 1.2 mg).

### Hematology and chemistry

EDTA treated whole blood was analyzed utilizing either an Abbott Cell-Dyn 3700 or Siemens Advia 120. Serum chemistries were conducted using Ortho Clincal Diagnostics Vitros 560. Blood was processed in accordance with blood tube manufacturer’s instructions.

### Statistical Analysis

Statistical testing was performed using GraphPad Prism software. More specifically, titers were log-transformed and a two-way Analysis of Variance (ANOVA) was performed with multiple comparisons.

### Ethics Statement

Animal research was conducted under an IACUC approved protocol at USAMRIID (USDA Registration Number 51-F-00211728 & OLAW Assurance Number A3473-01) in compliance with the Animal Welfare Act and other federal statutes and regulations relating to animals and experiments involving animals. The facility where this research was conducted is fully accredited by the Association for Assessment and Accreditation of Laboratory Animal Care, International and adheres to principles stated in the Guide for the Care and Use of Laboratory Animals, National Research Council, 2011.

## Data and materials availability

The data for this paper are included in either the paper or Supplemental Materials.

## Supplementary information

Supplementary Information.
