# Supplementary material for: Lipid Nanoparticle Formulation Increases Efficiency of DNA-Vectored Vaccines/Immunoprophylaxis in Animals Including Transchromosomic Bovines
Source: Sci Rep. 2020 May 29;10:8764. doi: 10.1038/s41598-020-65059-0 (PMC7260227; doi:10.1038/s41598-020-65059-0)
Supplement: Supplementary file 1 — Supplementary Information. [file 41598_2020_65059_MOESM1_ESM.pdf]

Title: Lipid Nanoparticle Formulation Increases Efficiency of DNA-Vectored Vaccines/Immunoprophylaxis in Animals Including Transchromosomic Bovines

Authors: Eric M. Mucker[1], Priya P.Karmali[2], Jerel Vega[2], Steven A. Kwilas[1], Hua Wu[4], Matthew Joselyn[1], John Ballantyne[3], Darryl Sampey[5], Rajesh Mukthavaram[2], Eddie Sullivan[4], Pad Chivukula[2], Jay W. Hooper[1*]

Supplemental method:

Transfection-based Potency Assay (for Table S1)

293T cells were plated at to be 60-90% confluent for the following day in a 12 well plate. The cells are in minimal essential media (MEM) containing 10% FBS at 37°C in a 5% CO_2 incubator.

The following day the transfection was setup using the standard FuGENE 6 protocol using the 6 to 1 ratio of reagent to DNA. For unformulated DNA FuGENE was added to Optimem for 5 min before DNA was added. After mixing with a pipet 10-20 times the mixture sat for 30min before it was added dropwise to the 293T cells with no additional change of media. Formulated DNA was added directly to the media in the wells. The plates incubated at 37°C in a 5% CO_2 incubator O/N.

The cells were manually detached from each well and mono suspended the following day and transferred to flow tubes. The cells were spun at 900g for 5min and washed twice with 3% normal goat serum in PBS. After the final wash the cells were blocked in the 3% NGS for 45min. Terminal rabbit bleed serum against ANDV or ZIKA was used at 1:400 for the primary antibody. After 1hr at 37C the cells were spun and washed 4x. A goat anti-rabbit 488 secondary (Invitrogen) at 1:500 was used as the secondary antibody. After 30 min at RT in a dark drawer the cells were spun and washed 4X. The cells were run on a BD FACScalibur cytometer, and analysis was conducted using FlowJo software.

Table S1. LNP-formulated DNA transfects 293T cells in the absence of a common (commercial) transfection reagent

pDNA	µg DNA	Percent of Positive cells (by flow cytometry)	
		Transfection Reagent	
		None	FuGENE 6
LNP-formulated ANDV	0.3	18.3	ND
ANDV	0.375*	3.3*	25.1
LNP-formulated ZIKV Lot A	0.6	76.5	ND
LNP-formulated ZIKV Lot B	0.6	79.2	ND
ZIKV Lot A	0.6	4.9	88.7
ZIKV Lot B	0.6	3.6	87.1
None	NA	1	3.2

NA= not applicable, ND= not done; pDNA= plasmid DNA; LNP= lipid nanoparticle

*1 µg when no transfection reagent used

Figure S1. Transient transfection of two cell lines to translate and secrete c7D11, a poxvirus specific monoclonal antibody, as determined by antigen specific ELISA. A, FuGENE 6 was utilized to transfect 293T cells in a T25 flask. The media was removed for testing and subsequently replaced. The accumulation of antibody up to and including the listed time point is given (µg). B, DNA was formulated with our LNP-carrier, serially diluted, and introduced to either Vero or 293T cell lines in a 24 well format. Supernatants were analyzed after three days.

A

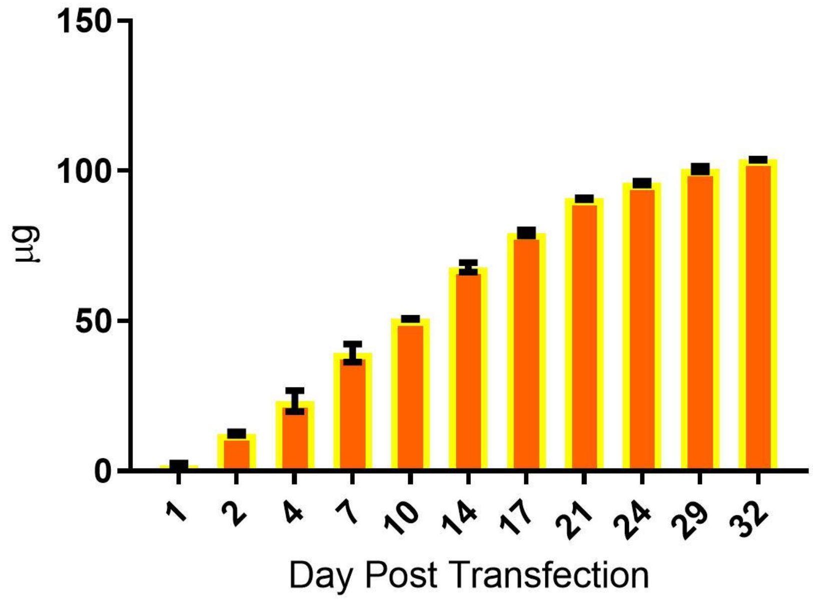

B

LNP Carrier?	Dilution (ug)	Cell line	ng/mL*
Yes	20	293T	ALOQ
	2		951.0
	0.2		275.4
	0.02		77.9
	0.002		9.1
	0.0002		4.2
Yes	4	Vero	11.4
	0.4		2.0
	0.04		1.6
	0.004		BLOQ
	0.0004		BLOQ
	0.00004		BLOQ

Figure S2. Cell blood counts of rabbits and nonhuman primates vaccinated with LNP formulated and unformulated DNA vaccine. Rabbits, A, and nonhuman primates, B, were vaccinated with the formulated or unformulated ANDES-M DNA. Nonhuman primates vaccinated with our Zika virus DNA vaccine are also provided, C.

A

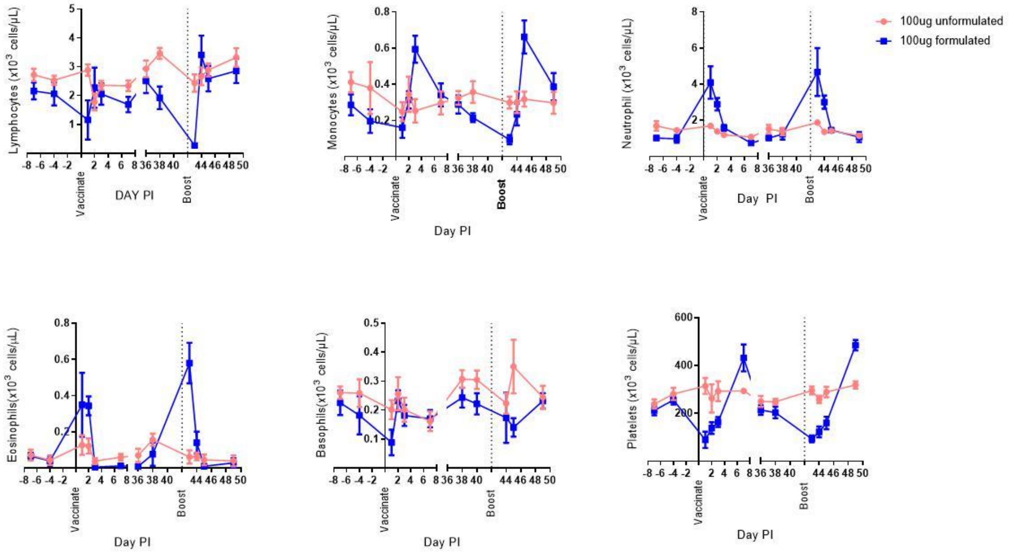

B

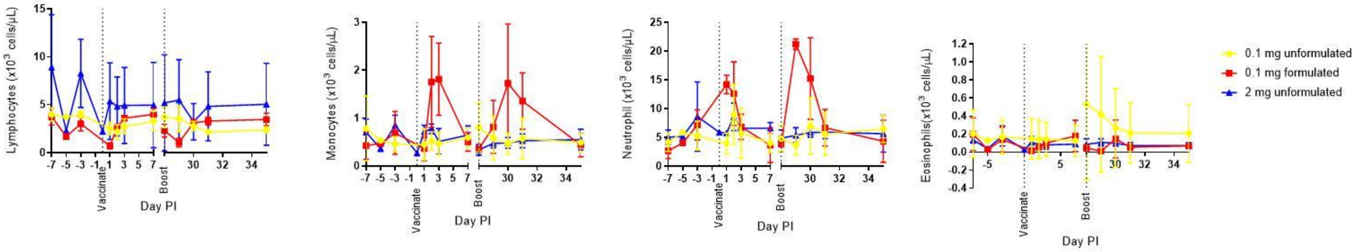

C

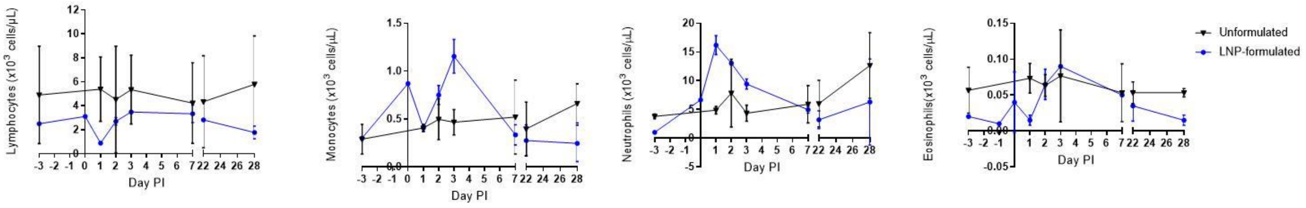

Figure S3. Light chain and heavy chain sequences for constructing a DNA plasmid capable of producing biologically (functionally) active, monoclonal antibody c7D11.

c7D11heavy chain

GCGGCCGCGCCACCATGAACCTGCTGCTCATTCTGACCTTTGTGGCCGCCGCTGTGGCCGAGGTGC
AGCTGGAGCAGAGCGGAGCCGAGCTCGCTAAGCCTGGCGCTAGCGTGAAGATGAGCTGCAAGGC
CTCCGGCTACACCTTCACCAGATACTGGATGCACTGGGTGAAGCAGAGACCCGGCCAGGGCCTGG
AATGGATCGGCTACATCAACCCCTCCACCGGCTACACCGAGTATAATCAGAAGTTCAAGGACAAG
GCCACACTCACCGCTGACAAATCCAGCTCCACCGTGTACATGCAGCTGTCCAGCCTCACAAGCGA
GGATTCCGCCGTGTACTACTGCGCCAGGACCACCGTGGACGGCTACGATTTCGCCTACTGGGGAC
AGGGCACCCTGGTGACCGTGAGCAGCGCTTCCACCAAGGGCCCCAGCGTGTTCCCTCTGGCTCCC
AGCTCCAAGAGCACATCCGGAGGCACCGCTGCTCTGGGCTGTCTGGTGAAGGACTACTTCCCTGA
GCCTGTGACCGTCAGCTGGAACAGCGGCGCTCTGACCAGCGGAGTGCACACCTTCCCTGCCGTCCT
GCAGAGCTCCGGCCTGTATAGCCTGAGCAGCGTCGTGACAGTGCCCTCCTCCAGCCTGGGAACCC
AGACATACATCTGCAACGTCAACCACAAGCCCAGCAACACCAAGGTGGACAAGAGAGTGGAACC
CAAGAGCTGTGACAAGACCCACACCTGCCCTCCCTGTCCCGCTCCTGAACTGCTGGGCGGACCCA
GCGTCTTTCTGTTCCCCCCCAAGCCCAAGGATACACTGATGATCAGCAGGACCCCCGAAGTGACCT
GCGTGGTGGTCGATGTCTCCCACGAGGACCCCGAGGTGAAGTTCAATTGGTATGTGGACGGCGTG
GAGGTGCATAATGCCAAAACCAAGCCCAGGGAAGAGCAGTACAACTCCACCTATAGAGTGGTGA
GCGTGCTGACAGTGCTGCACCAGGACTGGCTCAACGGCAAGGAGTACAAGTGCAAGGTGTCCAAC
AAAGCCCTCCCTGCCCCCATCGAGAAGACAATTTCCAAGGCCAAGGGACAGCCTAGAGAGCCCCA
GGTGTACACCCTGCCCCCCAGCAGAGAGGAGATGACCAAGAATCAGGTCTCCCTGACCTGCCTGG
TGAAGGGCTTCTACCCCTCCGATATCGCCGTGGAGTGGGAGAGCAACGGCCAGCCCGAGAACAAC
TACAAGACAACCCCCCCTGTGCTGGACAGCGACGGCTCCTTTTTCCTGTACAGCAAGCTGACCGTG
GACAAGAGCAGGTGGCAGCAAGGAAACGTCTTCTCCTGCTCCGTGATGCACGAGGCCCTCCACAA
TCACTACACACAGAAGAGCCTGAGCCTGAGCCCCGGCAAGTGATGAAGATCT

c7D11 light chain

GCGGCCGCGCCACCATGAACCTGCTGCTGATCCTGACCTTTGTGGCCGCCGCTGTGGCCGATATCG
TGATGAGCCAGAGCCCTAGCAGCCTGGCTGTGAGCGCCGGCGAGAAGGTGAGCATGAGCTGCAA
GAGCTCCCAGACCCTGCTGAACTCCAGGACCAGGAAGAACTACCTGGCCTGGTACCAGCAGAAGC
CTGGCCAGTCCCCTAAGCTCCTGATCTACTGGGCCAGCACAAGAGAGTCCGGCGTGCCCGACAGG
TTCACAGGCAGCGGAAGCGGCACCGACTTCACCCTGACCATCAGCAGCGTGCAGGCCGAGGACCT
CGCCGTGTACTACTGCAAGCAGTCCTACAACCTGTGGACCTTCGGCGGCGGCACCAAGCTGGAGA
TTAAGAGGACCGTCGCCGCCCCCAGCGTGTTTATCTTCCCCCCCAGCGACGAACAGCTGAAGAGC
GGAACCGCCAGCGTGGTGTGCCTGCTGAATAACTTCTACCCTAGGGAGGCCAAGGTGCAGTGGAA
GGTGGACAACGCCCTGCAGAGCGGCAACAGCCAGGAGTCCGTGACCGAGCAGGATAGCAAGGAC
TCCACCTACTCCCTGTCCAGCACCCTGACCCTGAGCAAGGCCGACTACGAGAAGCACAAGGTGTA
CGCTTGCGAGGTGACCCACCAGGGCCTGAGCTCCCCTGTGACCAAGTCCTTCAACAGGGGCGAGT
GCTGATGAAGATCT
